# Effects of intraoperative PEEP on postoperative pulmonary complications in patients undergoing robot-assisted laparoscopic radical resection for bladder cancer or prostate cancer: study protocol for a randomized controlled trial

**DOI:** 10.1186/s13063-019-3363-y

**Published:** 2019-05-29

**Authors:** Zhen-feng Zhou, Jun-biao Fang, Long Chen, Hong-fa Wang, Yong-jian Yu, Wen-yuan Wang, Jia-bao Chen, Miao-zun Zhang, Shuang-fei Hu

**Affiliations:** 1 Department of Anesthesiology, Zhejiang Provincial People’s Hospital (People’s Hospital of Hangzhou Medicine College), Hangzhou, 315000 China; 2 Department of General Surgery, Ningbo Medical Center Lihuili Hospital, Ningbo, 325000 China

**Keywords:** Positive end-expiratory pressure, Postoperative pulmonary complications, Robot-assisted surgery

## Abstract

**Background:**

There are increasing studies showing that the use of a lung-protective ventilation strategy has a lung protection effect in patients undergoing abdominal surgery; however, the appropriate positive end-expiratory pressure (PEEP) has not yet defined. Adopting a suitable PEEP may prevent postoperative pulmonary complications. Robot-assisted laparoscopic surgery is the newest and most minimally invasive treatment for bladder cancer or prostate cancer. It is also necessary to consider the effects of Trendelenburg position with pneumoperitoneum on airway pressure and pulmonary function. The role of PEEP during the intraoperative period in preventing postoperative pulmonary complications for robot-assisted laparoscopic surgery is not clearly defined.

**Methods/design:**

A total of 208 patients undergoing robot-assisted laparoscopic radical resection for bladder cancer or prostate cancer will be enrolled and then randomly assigned to a standard PEEP (6–8 cm H_2_O) group and a low PEEP (≤2 cm H_2_O) group. Both groups will receive an inspired oxygen fraction of 0.50 and a tidal volume of 8 mL/kg ideal body weight. Standard perioperative fluid management standardization and analgesic treatments will be applied in both groups. The primary endpoint is postoperative pulmonary complications within 7 days after surgery. Secondary endpoints are the modified clinical pulmonary infection score, postoperative extrapulmonary complications, postoperative surgical complications, intensive care unit length of stay, hospital length of stay, and 30-day mortality.

**Discussion:**

This trial aimed to assess the effects of low tidal volumes combined with intraoperative PEEP ventilation strategy on postoperative pulmonary complications in patients undergoing robot-assisted laparoscopic radical resection for bladder cancer or prostate cancer.

**Trial registration:**

ID: ChiCTR1800019867. Registered on December 2, 2018.

**Electronic supplementary material:**

The online version of this article (10.1186/s13063-019-3363-y) contains supplementary material, which is available to authorized users.

## Background

Robot-assisted laparoscopic surgeries, including robot-assisted laparoscopic radical prostatectomy (RALP) and robot-assisted laparoscopic radical cystectomy (RARC), are the newest and most minimally invasive treatments for bladder cancer or prostate cancer [1, 2]. The incidence of postoperative pulmonary complications (PPCs) in patients undergoing general surgery is about 5%, and 12% to 58% of patients undergoing abdominal surgery will develop a PPC [3, 4]. Furthermore, PPCs are strongly associated with prolonged postoperative hospital stays and a higher risk of mortality [5–7].

Nearly 30% of surgery patients undergoing general anesthesia and mechanical ventilation are at intermediate to high risk for PPCs, according to large cohort studies [4, 8]. Both alveolar overstretching and atelectasis induce the release of inflammatory mediators, leading to lung and systemic organ damage [9]. Lung-protective ventilation, including the use of low tidal volumes and positive end-expiratory pressure (PEEP), aims to prevent atelectasis and improve gas exchange [10, 11]. Furthermore, PEEP has been found to reduce mortality in patients with acute respiratory distress syndrome [12].

Adopting an appropriate PEEP may prevent PPCs. When high PEEP is applied, alveolar may be overinflated and pulmonary vascular resistance is likely to increase; however, use of low PEEP may not prevent atelectasis [9]. A number of studies have shown that, compared with non-protective mechanical ventilation without PEEP, the use of a lung-protective ventilation strategy has a lung-protective effect in patients with healthy lungs who are undergoing abdominal surgery, reducing the incidence of PPC [13, 14]. Although all of these studies recommend the use of a low tidal volume [9, 13–17], the appropriate PEEP has not yet been defined. A multicenter observational study showed that about 20% of patients did not receive PEEP during routine anesthetic practice [16]. In the Intraoperative Protective Ventilation (IMPROVE) trial, a lung-protective ventilation strategy with lower tidal volumes and a PEEP of 6 cm H_2_O was associated with improved clinical outcomes in patients undergoing major abdominal surgery [13]. Furthermore, a protective ventilation strategy with a PEEP of 10 cm H_2_O improved respiratory function and reduced the modified clinical pulmonary infection score (mCPIS) in another study including patients undergoing abdominal non-laparoscopic surgery [14]. However, another study showed that a low tidal volume combined with a low PEEP (3 cm H_2_O) ventilation may increase the risk of PPCs during major surgery such as hepatectomy [17]. In the PROVHILO trial, a ventilation strategy of high PEEP (12 cm H_2_O) did not reduce the incidence of PPCs but more likely caused hemodynamic instability in patients undergoing open abdominal surgery [15]. Therefore, the authors suggested a ventilation strategy of a low tidal volume combined with a low PEEP (≤2 cm H_2_O).

It should be noted that these studies included only open surgeries or various types of abdominal surgery; they did not include patients planning to undergo robot-assisted laparoscopic surgery. Furthermore, it is necessary to consider the effects of steep Trendelenburg (sT) positioning and pneumoperitoneum (PnP) on airway pressure and pulmonary function [18], which can increase intra-abdominal pressure and enhance the cranial displacement of the diaphragm. This displacement will decrease lung compliance and lung volumes and increase lung resistance. Adopting a PEEP of 7 cm H_2_O during RALP, which could improve arterial oxygenation without causing excessive peak airway pressure, has been recommended [19]. Also, a lung-protective ventilation strategy with a lower tidal volume (V_t_) of 6 mL/kg and a PEEP of 8 cm H_2_O was associated with less impaired postoperative pulmonary functions in patients undergoing RALP in another recent study [20].

The role of PEEP during the intraoperative period in preventing PPCs for robot-assisted laparoscopic surgery has not been clearly defined. This study may further improve our knowledge regarding the effects of intraoperative PEEP on PPCs, survival rates, and in-hospital stays in patients undergoing robot-assisted laparoscopic radical resection for bladder cancer or prostate cancer.

## Methods/design

### Objectives of the study

This trial aimed to compare the effects of low tidal volumes combined with standard PEEP (6–8 cm H_2_O) with those of low PEEP (≤2 cm H_2_O) in patients at risk for complications undergoing robot-assisted laparoscopic radical resection for bladder cancer or prostate cancer during general anesthesia in terms of (1) PPCs; (2) mCPIS, postoperative extrapulmonary complications, changes in chest x-ray findings, and oxygenation; (3) intraoperative complications, including hypoxemia, hypotension, and massive transfusion; and (4) postoperative surgical complications, intensive care unit (ICU) lengths of stay, hospital lengths of stay, and 30-day mortality.

### Study design

**Fig. 1:**
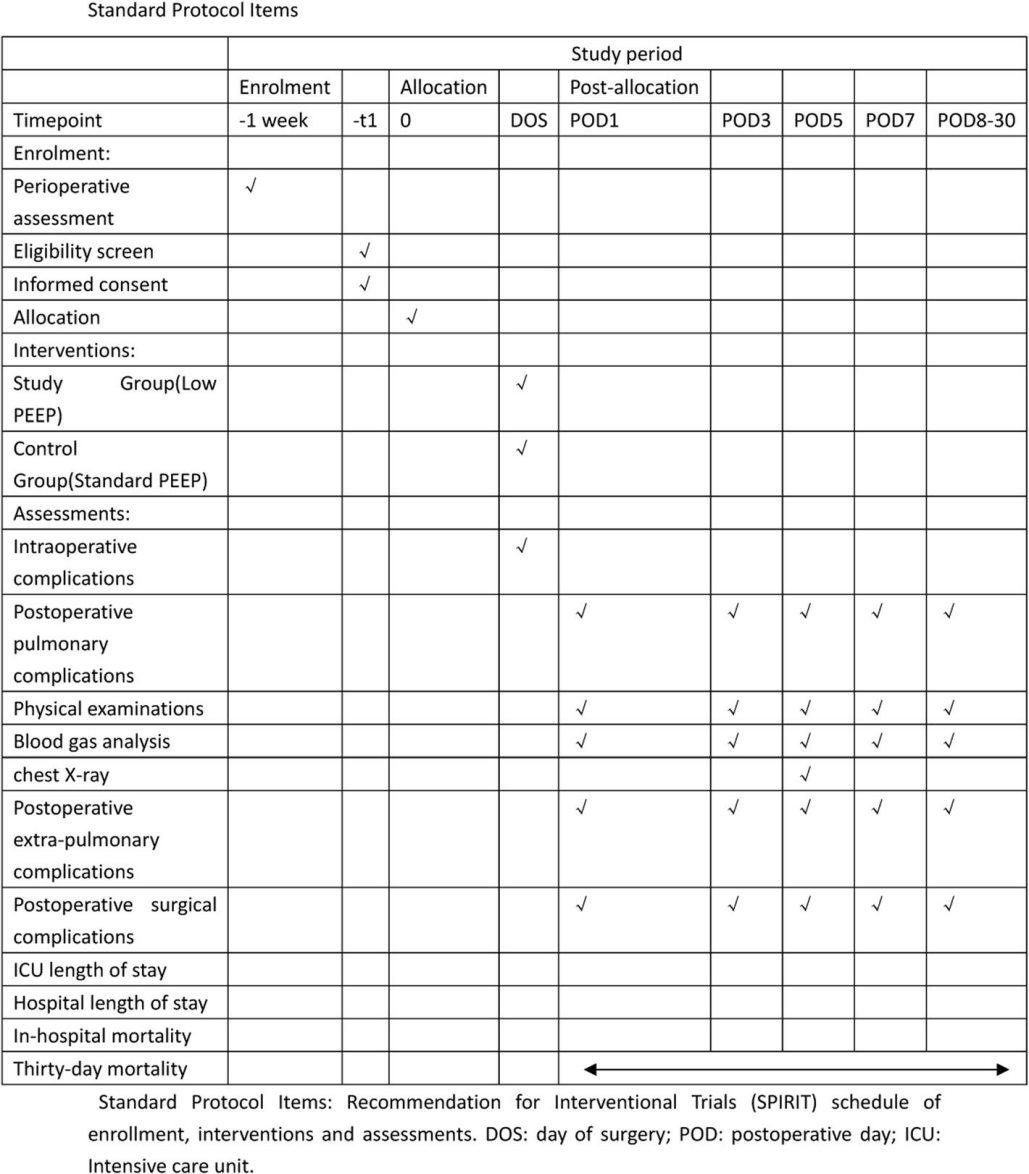
Standard protocol items

This unfunded, parallel-group, double-blinded, prospective, randomized controlled clinical trial was registered at http://www.chictr.org.cn (ChiCTR1800019867) and was conducted at the Department of Anesthesiology and Intensive Care of Zhejiang Provincial People’s Hospital. The first patient will be randomly assigned in January 2019. This trial protocol is conducted in accordance with the Consolidated Standards of Reporting Trials (CONSORT) guidelines (Fig. 1). The SPIRIT (Standard Protocol Items: Recommendation for Interventional Trials) 2013 Checklist is presented in Additional file 1.

### Blinding, data collection, randomization, and record-keeping

#### Selection of the participants

Researchers will be trained prior to investigation. Study data, including patient clinical characteristics, intraoperative respiratory parameters, postoperative outcomes, and laboratory tests, will be recorded in case report forms (CRFs) (Additional file 2).

An independent researcher will randomly assign the participants to the study group (standard PEEP group) and control group (low PEEP group) in a ratio of 1:1. The random sequence will be computer-generated, and participants will be allocated in numerical order with sealed opaque envelopes. The attending anesthesiologist performs anesthesia strictly in accordance with the research protocol and is also responsible for data during the preoperative, intraoperative, and post-anesthesia care unit (PACU) period. The chief surgeon performs the postoperative laboratory testing. An independent researcher will be involved in postoperative follow-up and data collection. Statistical analysis will be performed by a statistician who does not participate in the data collection. Patients, research staff, surgeons, ICU physicians, and the statistician will be unaware of the group allocation. Some preoperative characteristics and laboratory results will be automatically derived from a computer database.

The original data (CRF and relevant records) will be maintained for 10 years and then destroyed in accordance with hospital standards.

#### Selection of the participants

Patients scheduled for elective robot-assisted laparoscopic radical resection for bladder cancer or prostate cancer under general anesthesia will be screened and recruited during preoperative assessment. Patients meeting inclusion criteria will be required to provide written informed consent. The participant can withdraw from the trial at any time.

Inclusion criteria are patients older than 18 years, American Society of Anesthesiologists (ASA) physical status I–III, and body mass index (BMI) between 18 and 35 kg/m^2^. Exclusion criteria are listed as following: emergency surgery or history of lung surgery; history of mechanical ventilation within the 2 weeks before recruitment; non-invasive ventilation or oxygen therapy at home; acute respiratory failure (pneumonia, acute lung injury, or acute respiratory distress syndrome); history of chronic obstructive pulmonary disease, persistent hemodynamic instability, or severe cardiac disease (New York Heart Association class III or IV, persistent ventricular tachyarrhythmias, or acute coronary syndrome); sepsis or septic shock; need for continuous renal replacement therapy; progressive neuromuscular illness; pregnancy; participation in another study; or refusal to participate.

### Time course of the study

#### Preoperative admission

Medical history, ASA physical status, BMI, 12-lead electrocardiogram (ECG), laboratory results, chest x-ray or computed tomography (CT) scan, ARISCAT (Assess Respiratory Risk in Surgical Patients in Catalonia) score (the ARISCAT study; Additional file 3), nutritional risk screening (NRS 2002 tool), and the results of echocardiography and spirometry (in cases of history of coronary artery disease or smoking) will be recorded.

#### Intraoperative care

A central venous catheter and an arterial cannula will be placed before induction of anesthesia. Peripheral oxygen saturation (SpO_2_), arterial blood pressure, heart rate, ECG, end-tidal carbon dioxide tension (EtCO_2_), and bispectral index (BIS) will be monitored continuously. PnP, tidal volume, PEEP, airway pressures including peak pressure and plateau pressure, airway resistance (Raw), dead space fraction (Vds/Vt), core temperature, and arterial blood gas analysis data will be recorded.

Crystalloid (12–15 mL/kg per hour) is infused to maintain hemodynamic stability and a central venous pressure of 5–12 cm H_2_O. Blood loss and vasodilation are supplemented by colloidal fluid.

Routine anesthesia is induced with intravenous dexmedetomidine (1 μg/kg) or midazolam (0.05–0.075 mg/kg), cisatracurium (2 mg/kg), propofol (2–3 mg/kg), and fentanyl (1–3 μ/kg) for tracheal intubation. Anesthesia is maintained with propofol, sevoflurane, and remifentanil infusion to maintain the BIS of 40–50 until skin suturing is completed. Cisatracurium (1.0–1.5 mg/kg) is administered every hour and is adjusted in accordance with the anesthesiologist’s decision.

Ropivacaine is administrated as local anesthetic before and at the end of operation. Fentanyl (1–3 μg/kg) and flurbiprofenaxetil 50 mg are required before remifentanil is stopped.

#### Postoperative care

Patients will be transferred to the PACU after surgery regardless of whether they are still intubated. Postoperative pain management will be suggested to achieve a visual analogue scale (VAS) pain score of less than 3 out of 10 using a patient-controlled intravenous analgesia pump including fentanyl (0.3–0.5 μg/kg), flurbiprofenaxetil (100 mg), and palonosetron hydrochloride (0.25 mg) palazidine.

The ICU physician and surgeon will independently monitor clinical progress and all endpoints by daily physical examinations. Appropriate prophylactic antibiotics and antithrombotic treatments will be administered as required during the postoperative period. Chest x-ray or CT scanning will be performed when clinically indicated. Arterial blood gas analysis will be performed on postoperative day 1 (POD 1) and POD 3, and other laboratory tests will be performed on POD 1, POD 3, POD 5, and POD 7. The examinations will be repeated and microbiology tests will be performed when the development of pulmonary complications is suspected.

#### Study arms and intraoperative ventilation protocol

Patients will be randomly assigned to the low PEEP ventilation group (PEEP ≤ 2 cm H_2_O) or the standard PEEP group (PEEP = 6–8 cm H_2_O) using a volume-controlled ventilation strategy (Datex Ohmeda S/5 Avance; GE Healthcare, Helsinki, Finland) with a tidal volume of 8 mL/kg ideal body weight (IBW), an inspired oxygen fraction (FiO_2_) of 0.50, and an inspiratory-to-expiratory ratio of 1:2. Respiratory rate should be adjusted to maintain EtCO_2_ between 35 and 45 mm Hg, and plateau pressure should be no more than 30 cm H_2_O. IBW is calculated as follows [13]: 45.5 + 0.91 × (centimeters of height − 152.4) for females and 50 + 0.91 × (centimeters of height − 152.4) for males. Recruitment maneuvers (RMs) [21] will be performed immediately after tracheal intubation and every time the ventilator is interrupted until the end of surgery in each group. The compliance of the respiratory system will be calculated as V_t_ / (plateau pressure of the respiratory system − PEEP).

RMs will be performed as follows:

1. Pressure support ventilation (PSV) mode
2. PEEP set to 30 cm H_2_O
3. Inspiratory gas flow set to the highest value
4. Duration of the maneuver = 30 s.

A rescue therapy will be applied in case of desaturation (defined as a peripheral SpO_2_ of less than 92%), consisting of increased FiO_2_ to 100% in each group and increasing PEEP in the low PEEP group (Additional file 4).

### Study endpoints

#### Primary outcome measure

The primary endpoint is PPCs, including new atelectasis or infiltrates on a chest x-ray or CT scanning, respiratory failure defined as the need for non-invasive or invasive ventilation or partial pressure of arterial oxygen/fraction of inspired oxygen (PaO_2_/FiO_2_) of less than 300 within 7 days after surgery [21].

#### Secondary outcome measures

Secondary outcome variables are any pulmonary complications and extrapulmonary complications as follows (Additional file 5):

1. Intraoperative complications: pneumothorax confirmed by chest x-ray and any other complications.
2. PPCs within 30 days after surgery. Those PPCs are scored in accordance with a grading scale ranging from 0 to 4 [22] (grade 0 representing no PPCs and grades 1 to 4 representing gradually worse forms of PPCs) within 7 and 30 days after surgery (Table 1).
3. PPCs will also be analyzed separately (Table 1).
  - Pneumonia is defined in accordance with criteria of the Centers for Disease Control and Prevention [23];
  - Purulent sputum;
  - Postoperative hypoxemia and severe hypoxemia [24];
  - Suspected pulmonary infection is described in a previous study [15];
  - Pulmonary infiltrate is defined in accordance with consensus guidelines [25]: chest x-ray demonstrating monolateral or bilateral infiltrate.
  - Atelectasis, pleural effusion, and pneumothorax are identified by chest x-ray.
  - The mCPIS is calculated as previously described [26] (Table 2).
  - Suspected pulmonary complications [14];
  - Requirement for postoperative ventilation (respiratory failure that requires non-invasive or invasive ventilation or both) at any time after surgery in accordance with standard criteria and clinical practice guidelines [22].
4. Postoperative extrapulmonary complications within 30 days after surgery:
  - Criteria for systemic inflammatory response syndrome [12]
  - Sepsis and severe sepsis [12]
  - Septic shock [12]
  - Other extrapulmonary infection including surgical site infection (SSI) [27] and intra-abdominal abscess
  - Need for postoperative blood transfusion
  - Postoperative surgical complications: anastomotic leakage and need for surgical re-intervention, defined in accordance with consensus criteria [28]
  - Unexpected ICU admission or readmission
  - ICU length of stay and hospital length of stay
  - Hospital-free days at follow-up day 30
  - In-hospital mortality and 30-day mortality (all-cause mortality 30 days after randomization).

**Table 1:** Grade scale for postoperative pulmonary complications

Grade scale	Detailed description
Grade 1	- Cough, dry - Microatelectasis: abnormal lung findings and temperature greater than 37.5 °C without other documented cause; results of chest radiograph indicates no new findings as compared to pre-operative result - Dyspnea, not due to other documented cause
Grade 2	- Cough, productive, not due to other documented cause - Bronchospasm: new wheezing or pre-existent wheezing resulting in change of therapy - Hypoxemia - Atelectasis: radiological confirmation plus either a temperature of more than 37.5 °C or abnormal lung findings - Hypercarbia, transient, requiring treatment, such as naloxone or increased manual or mechanical ventilation
Grade 3	- Pleural effusion, resulting in thoracentesis - Pneumonia, suspected: radiological evidence without bacteriological confirmation - Pneumonia, proven: radiological evidence and documentation of pathological organism by Gram stain or culture - Pneumothorax - Re-intubation postoperative or intubation, period of ventilator dependence (non-invasive or invasive ventilation) of not more than 48 h
Grade 4	Ventilatory failure: postoperative non-invasive ventilation dependence of at least 48 h or re-intubation with subsequent period of ventilator dependence of at least 48 h

**Table 2:** The definition of modified clinical pulmonary infection score

Items	CPIS points		
	0	1	2
Tracheal secretions	Rare	Abundant	Abundant + purulent
Chest x-ray infiltrates	No infiltrate	Diffused	Localized
Temperature, °C	36.5–38.4	38.5–38.9	≤36.5 or ≥39.0
Leukocyte count, per mm^3^	4000–11,000	<4000 or >11,000	<4000 or >11,000 + band forms ≥500
PaO_2_/FiO_2_, mm Hg	>240 or ARDS		≤240 and no evidence of ARDS
Microbiology	Negative		Positive

*Abbreviations*: *ARDS* Acute respiratory distress syndrome, *CPIS* clinical pulmonary infection score, *PaO*_*2*_*/FiO*_*2*_ Partial pressure of arterial oxygen/fraction of inspired oxygen

### From postoperative day 7 (POD 8 to POD 30, follow-up)

**Fig. 2:**
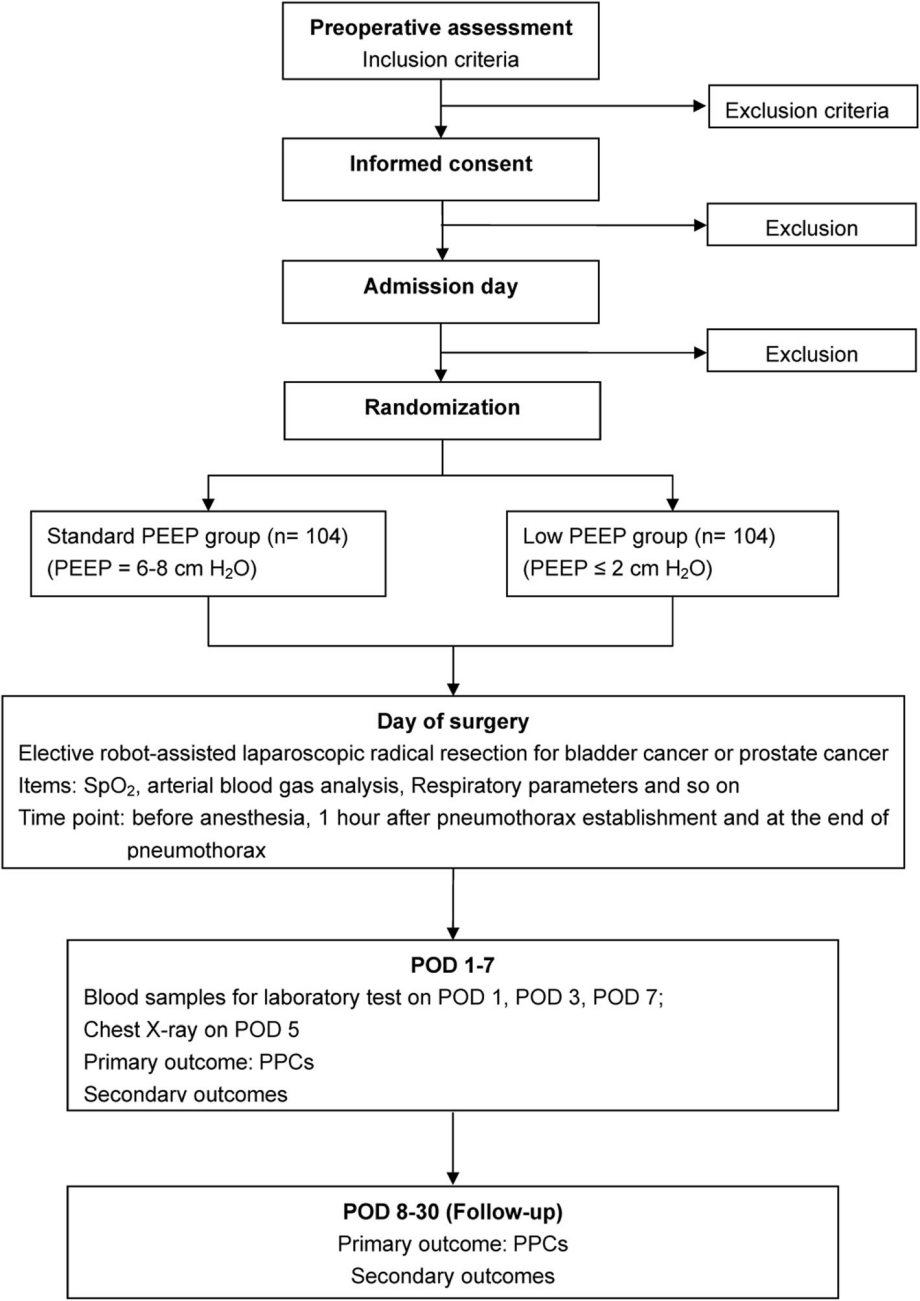
The CONSORT (Consolidated Standards of Reporting Trials) flowchart of the trial

Secondary endpoints and any mortality will also be evaluated during the follow-up period. The CONSORT flowchart of the trial is shown in Fig. 2.

### Data monitoring and handling of implausible values or missing values

A clinical investigator will identify implausible values. Missing continuous variables should be less than 10% and will be replaced by median. Missing values will be replaced by the mean of all plausible data (both groups) of the respective endpoint. Data monitoring is managed by an independent investigator who is not involved in the study. The progress of the study will be evaluated, and the completeness and accuracy of the data (informed consent forms, source data, CRFs, and outcome variables) will be verified.

### Statistics

Normally distributed variables will be expressed as the mean ± standard deviation and will be compared with the Student’s *t* test. Categorical variables will be compared by using the chi-squared test or Fisher’s exact test. Non-normal continuous variables will be expressed as median (interquartile range) and evaluated with the Mann–Whitney *U* test. Intention-to-treat analyses are performed to compare the composite outcome measure at 7 days in the two groups by the chi-squared test (or Fisher’s exact test as appropriate), and multiple logistic regression analysis adjusting will be performed to identify various risk factors (for the primary outcome and the pulmonary complications at POD 30). Adjusted analyses are performed with the use of robust Poisson generalized-linear-model regression for continuous outcomes and are presented as relative risks with 95% confidence intervals. A *P* value of less than 0.05 will be considered statistically significant, and all reported *P* values will be two-sided. Interim analysis of safety will be conducted after enrollment of the first 200 patients. All analyses will be conducted by using SPSS version 18.0 (SPSS, Chicago, IL, USA) software.

### Sample size calculation

The incidence rate of PPCs is 39% in the low PEEP group [15]. The two-tailed chi-squared test is performed, and we estimate that 188 patients are required to provide 90% power to detect a 50% relative difference between the two groups (39% to 20%), and type I error probability was 0.05. Given that the follow-up lost rate is 10%, 208 cases in total are needed. Analysis is computed by using G-Power (version 3.1; Informer Technologies, Inc. Universität Kiel, Germany).

### Adverse events and interruption of the trial

All patients will be continuously monitored during the study, including daily visits during in-hospital and daily phone-call visits during the out-of-hospital follow-up period (until POD 30). All serious adverse, unexpected, or possibly related events will be recorded in the CRF and will be reported to the data monitoring and safety committee (DMSC). DMSC can recommend that the study should be stopped unless there is evidence that patient is safety (a between-group difference in serious adverse events or in 30-day mortality is found).

## Discussion

In this pragmatic, prospective, randomized controlled trial of patients undergoing robot-assisted laparoscopic radical resection for bladder cancer or prostate cancer, our aim will be to assess not only possible single effects of PEEP levels on major PPCs from those of lower tidal volumes and RM but also relevant clinical parameters associated with alterations in pulmonary function, such as chest x-ray, abnormalities, mCPIS, arterial oxygenation/peripheral oxygen saturation in air, and changes in dyspnea/cough/secretions. Our findings might change the current practice of mechanical ventilation in patients undergoing robot-assisted laparoscopic radical resection for bladder cancer or prostate cancer.

Notably, mechanical ventilation itself is one of the major contributors to PPCs [29]. sT positioning together with pneumoperitoneum is also an important risk factor for PPCs [30]. Intra-abdominal pressure is frequently higher than airway pressure during PnP with carbon dioxide (CO_2_) for laparoscopic surgery. This pressure gradient usually causes cephalad displacement of the diaphragm and collapses adjacent pulmonary tissues. PnP also decreases respiratory compliance and arterial oxygenation [31]. All of these influences on PnP lead finally to atelectasis [32]. The major difference between robot-assisted surgeries and other laparoscopic surgeries is the sT positioning, which will further decrease respiratory compliance and vital capacity.

On the other hand, PEEP is thought to prevent the development of atelectasis by keeping the airways open and maintaining adequate gas exchange at the end of the expiratory period during PnP [9]. Certainly, the level of PEEP should be adopted according to the patient’s and surgical characteristics as well as to the patient’s positioning.

The optimal PEEP has not yet been defined in patients undergoing robot-assisted laparoscopic surgery, even though adopting a PEEP of over 5 cm H_2_O in patients undergoing laparoscopic surgery is recommended [11]. One study recommended adopting a PEEP of 7 cm H_2_O during RALP [19], and another recent study found that a PEEP of 8 cm H_2_O was the optimal level of PEEP in patients undergoing RALP [20]. As we know that low levels of PEEP are potentially associated with atelectasis by promoting repeated opening and closing of small airways [33]. However, higher levels of PEEP may increase mean airway pressure of the respiratory system and likely even impair hemodynamics.

There is an increasing number of highly qualitative randomized controlled trials regarding intraoperative mechanical ventilation and PPCs in both abdominal surgeries [10, 11] and laparoscopic surgeries [32], whereas direct assessment of the effect in patients undergoing robot-assisted laparoscopic radical resection for bladder cancer or prostate cancer is still lacking. The potential significance of this trial is that it may provide evidence of the effects of intraoperative PEEP on PPCs in patients undergoing robot-assisted laparoscopic radical resection for bladder cancer or prostate cancer.

There are some potential strengths of the present trial protocol. First, the included patients will undergo elective robot-assisted laparoscopic radical resection for bladder cancer or prostate cancer with longer anesthesia duration, which is a potential risk factor for PPCs [7]. Second, this trial design includes instructions for fluid management standardization and analgesic treatments during the perioperative period. Third, the adopting ARISCAT score is considered the most valuable tool for predicting PPCs, although various scores have been developed for predicting PPC incidence based on various countries and surgical populations [9].

## Trial status

The study protocol version number is V1.0 (September 10, 2018). It is first submitted to the ethics committee of Zhejiang Provincial People’s Hospital (People’s Hospital of Hangzhou Medicine College) on September 10, 2018, and finally approved on October 22, 2018 (Additional files 6 and 7). The first participant is expected to be recruited before March 2019, and the estimated completion date of recruitment is October 2021.

## Additional files

Additional file 1: SPIRIT (Standard Protocol Items: Recommendation for Interventional Trials) 2013 Checklist: Recommended items to address in a clinical trial protocol and related documents. (DOC 123 kb)

Additional file 2: Case report form. (DOC 274 kb)

Additional file 3: Preoperative risk index of postoperative pulmonary complications by ARISCAT (Assess Respiratory Risk in Surgical Patients in Catalonia) score. (DOC 40 kb)

Additional file 4: Strategy for oxygen saturation (SpO_2_) decreasing. (DOC 28 kb)

Additional file 5: Definitions of study endpoints. (DOC 42 kb)

Additional file 6: Appendix Figure study institutional review board (IRB) in original language. (TIF 390 kb)

Additional file 7: Appendix Figure study institutional review board (IRB) in English. (TIF 401 kb)

## Abbreviations

ARISCAT: Assess Respiratory Risk in Surgical Patients in Catalonia
ASA: American Society of Anesthesiologists
BIS: Bispectral index
BMI: Body mass index
CONSORT: Consolidated Standards of Reporting Trials
CRF: Case report form
CT: Computer tomography
DMSC: Data monitoring and safety committee
ECG: Electrocardiogram
EtCO_2_: End-tidal carbon dioxide tension
FiO_2_: Fraction of inspired oxygen
IBW: Ideal body weight
ICU: Intensive care unit
mCPIS: Modified clinical pulmonary infection score
PACU: Post-anesthesia care unit
PEEP: Positive end-expiratory pressure
PnP: Pneumoperitoneum
POD: Postoperative day
PPC: Postoperative pulmonary complication
RALP: Robot-assisted laparoscopic radical prostatectomy
RM: Recruitment maneuver
SpO_2_: Oxygen saturation
sT: Steep Trendelenburg
V_T_: Tidal volume
