# Supplementary material for: Effects of intraoperative PEEP on postoperative pulmonary complications in patients undergoing robot-assisted laparoscopic radical resection for bladder cancer or prostate cancer: study protocol for a randomized controlled trial
Source: Trials. 2019 May 29;20:304. doi: 10.1186/s13063-019-3363-y (PMC6542052; doi:10.1186/s13063-019-3363-y)
Supplement: Supplementary file 2 — Case report form. (DOC 274 kb) [file 13063_2019_3363_MOESM2_ESM.doc]

**Effects of intraoperative PEEP on postoperative pulmonary complications in patients undergoing robot-assisted laparoscopic radical resection for bladder cancer or prostate cancer: study protocol for a randomized controlled trial**

# Case report form

# Checklist

**Please check the checklist carefully when completed the questionnaire.**

□**Perioperative assessment: Informed patient details of the study and obtained informed consent from the patient.**

**Date of signing informed consent:**

**Admission date:**

□**Perioperative data Surgery date:**

□**Completed the above registrations**

**Completed date:**

**Quality control personnel：________**

# Case registration form

## Basic information

**Case number:**

**Patient case number:**

**Random number:**

**Randomized group：🞏 Low-PEEP group (Study Group)**

**🞏 Standard PEEP group (Control Group)**

**Gender male/female**

**Height _________________________cm**

**Ideal weight _________________________kg**

**Actual weight _________________________kg**

Calculation formula of ideal weight：

male= 50 + 0.91×(Height - 152.4)；

female= 45.5±0.91×(Height - 152.4)

**Age (years) _____**

**Contact staff _________________________**

**Phone number _______________________**

**Address ___________________________**

**Chief surgeon ______________________**

**Baseline Characteristics of the Patients**

**1. Inclusion and exclusion criteria** (Please √ if there is any situation as listed below)

| **Item** | **Inclusion criteria** | |
| --- | --- | --- |
|  | **yes** | **no** |
| Age：≥ 18 year |  |  |
| Scheduled for elective robot-assisted laparoscopic radical resection for bladder cancer or prostate cancer |  |  |
| ASA physical status I-III |  |  |
| BMI: 18-35 kg/m^2^ |  |  |
|  | **Exclusion criteria** | |
| Emergency surgery |  |  |
| Mechanical ventilation of > 1 hour within the last 2 weeks before surgery |  |  |
| History of previous severe (COPD) |  |  |
| Acute respiratory failure (pneumonia, acute lung injury or acute respiratory distress syndrome) |  |  |
| Previous lung surgery |  |  |
| Persistent hemodynamic instability or Severe cardiac disease |  |  |
| Sepsis or septic shock |  |  |
| Need renal replacement therapy |  |  |
| Progressive neuromuscular illness |  |  |
| Pregnancy |  |  |
| Consented for another interventional study or refusal to participate |  |  |
| Have a stake in the researcher |  |  |
| Researchers conside that they are not suitable for clinical trials |  |  |

**2. Perioperative data** (Please √ or Write down specific situation or if there is any situation as listed below and please / If there is no corresponding situation)

| **Preoperative** |  | **One hour after pneumothorax establishment** |  | **at the end of pneumothorax** |  |
| --- | --- | --- | --- | --- | --- |
| Temperature |  | arterial blood gas analysis |  |  |  |
| ASA status |  | FiO_2_ |  |  |  |
| ARISCAT score |  | Pneumoperal pressure |  |  |  |
| NYHA III-IV |  | Tidal volume |  |  |  |
| History of smoking |  | Respiratory rate |  |  |  |
| Drinking |  | PT-CO_2_ |  |  |  |
| Combined diseases |  | PEEP |  |  |  |
| History of Medication |  | Platform / peak pressure |  |  |  |
| Respiratory infection within one month |  | Blood pressure |  |  |  |
| Weight change in the past one month |  | Heart rate |  |  |  |
| Blood routine  examination |  | Temperature |  |  |  |
| Coagulation spectrum |  |  | | | |
| Biochemical tests |  | **Intra-operative** |  |  |  |
| Chest X-ray or CT |  | Times of RM |  |  |  |
| Pulmonary function test |  | Adverse events during RM |  |  |  |
| mCPIS score |  | Antibiotic |  |  |  |
|  |  | Infusion volume |  |  |  |
|  |  | Blood transfusion |  |  |  |
| **Before anesthesia** |  | Amount of bleeding |  |  |  |
| SpO_2_ without inhaling oxygen |  | Urine volume |  |  |  |
| Arterial blood gas analysis |  | Vasoactive drug use |  |  |  |
|  |  | Operation time |  |  |  |
|  |  | Mechanical ventilation time |  |  |  |
|  |  | Other complications |  |  |  |

The amount of each day and duration for smoking and drinking; describe the specific disease and current medication and doses for columns of combined disease and medication history; only check if there is laboratory test or chest X-ray. etal; arterial blood gas analysis should performed after10 min of air adaptation before anesthesia;

Intraoperative complications were recorded and defined as follows: 1. peripheral oxygen saturation less than 90% and/or end-tidal fractions of carbon dioxide more than 45 mmHg for more than 1 min, 2. need to change the ventilation setting (tidal volume and/or respiratory rate), 3. heart rate more than 100 beats/min or less than 60 beats/min, 4. systolic arterial pressure more than 150 mmHg or less than 90 mmHg.

Blood gas analysis during postoperative recovery room should done meet the following 2 points at the same time: 1. 30 minutes after the tracheal tube is removed; 2. after10 min of air adaptation. If peripheral oxygen saturation dropped below 88% during the 10 min of adaptation, the maneuver was stopped and arterial blood gas analysis immediately obtained.

**3. Postoperative pulmonary complications within 30 days after surgery** (Please √ or Write down specific situation or if there is any situation as listed below and please / If there is no corresponding situation)

| **Items/times** | **POD 1** | **POD 3** | **POD 5** | **POD 7** | **POD 8-30** |
| --- | --- | --- | --- | --- | --- |
| SpO_2_ after10 min of air adaptation |  |  |  |  | ／ |
| FiO_2_ after10 min of air adaptation |  |  |  |  | ／ |
| Arterial blood gas analysis |  |  | ／ | ／ | ／ |
| Heart rate |  |  |  |  |  |
| Respiratory rate |  |  |  |  |  |
| Chest X-ray | ／ | ／ |  | ／ |  |
| Blood routine test |  |  |  |  |  |
| CRP |  |  |  |  |  |
| Biochemical tests |  |  |  |  |  |
| Microbiology test |  |  |  |  |  |
| Mechanical Ventilation |  |  |  |  |  |
| Whether tracheal secretions was increased; the nature and quantity of secretions |  |  |  |  |  |
| Cough |  |  |  |  |  |
| Difficulty breathing |  |  |  |  |  |
| Chest pain |  |  |  |  |  |
| Postoperative hypoxemia |  |  |  |  |  |
| Postoperative severe hypoxemia |  |  |  |  |  |
| Suspected lung infection |  |  |  |  |  |
| Pneumonia |  |  |  |  |  |
| Exudation of the lungs |  |  |  |  |  |
| Aspiration pneumonia |  |  |  |  |  |
| Pulmonary embolism |  |  |  |  |  |
| Atelectasis |  |  |  |  |  |
| ALI/ARDS |  |  |  |  |  |
| Pneumothorax |  |  |  |  |  |
| Pleural effusion |  |  |  |  |  |

ALI: Acute lung injury; ARDS: acute respiratory distress syndrome.

**3. Postoperative extra-pulmonary complications within 30 days after surgery** (Please √ or Write down specific situation or if there is any situation as listed below and please / If there is no corresponding situation)

| **Items/times** | **POD 1** | **POD 3** | **POD 5** | **POD 7** | **POD 8-30** |
| --- | --- | --- | --- | --- | --- |
| SIRS |  |  |  |  |  |
| Sepsis |  |  |  |  |  |
| Severe sepsis |  |  |  |  |  |
| Sepsis shock |  |  |  |  |  |
| Extrapulmonary infection |  |  |  |  |  |
| Acute myocardial infarction |  |  |  |  |  |
| Pulmonary edema caused by heart failure |  |  |  |  |  |
| Coma |  |  |  |  |  |
| AKI |  |  |  |  |  |
| DIC |  |  |  |  |  |
| Blood transfusion |  |  |  |  |  |
| Anastomotic leak |  |  |  |  |  |
| Secondary surgery rate |  |  |  |  |  |

SIRS: Systemic inflammatory response syndrome; AKI: acute kidney injury; DIC: disseminated intravascular coagulation.
