# Supplementary material for: Effects of intraoperative PEEP on postoperative pulmonary complications in patients undergoing robot-assisted laparoscopic radical resection for bladder cancer or prostate cancer: study protocol for a randomized controlled trial
Source: Trials. 2019 May 29;20:304. doi: 10.1186/s13063-019-3363-y (PMC6542052; doi:10.1186/s13063-019-3363-y)
Supplement: Supplementary file 3 — Preoperative risk index of postoperative pulmonary complications by ARISCAT (Assess Respiratory Risk in Surgical Patients in Catalonia) score. (DOC 40 kb) [file 13063_2019_3363_MOESM3_ESM.doc]

| Additional file 3: preoperative risk index of postoperative pulmonary complications by ARISCAT score | | |
| --- | --- | --- |
| Preoperative risk factor |  | Point Valve |
| Age (year) |  |  |
| ≤ 50 |  |  |
| 51–80 |  | 3 |
| > 80 |  | 16 |
| 50-59 |  | 4 |
| Preoperative SpO_2_ (%) |  |  |
| ≥ 96 |  |  |
| 91–95 |  | 8 |
| ≤ 90 |  | 24 |
| Respiratory infection in the last month |  | 17 |
| Preoperative anemia (≤ 100 g/L) |  | 11 |
| Surgical incision |  |  |
| Peripheral |  |  |
| Upper abdominal |  | 15 |
| Intrathoracic |  | 24 |
| Duration of surgery (h) |  |  |
| ≤ 2 |  |  |
| 2 - 3 |  | 16 |
| > 3 |  | 23 |
| Emergency procedure |  | 8 |
| Intermediate Risk= 26–44 Points; High Risk= ≥45 Points; SpO_2_= oxyhemoglobin saturation by pulse oximetry breathing air in supine position. | | |
