# Supplementary material for: Effects of intraoperative PEEP on postoperative pulmonary complications in patients undergoing robot-assisted laparoscopic radical resection for bladder cancer or prostate cancer: study protocol for a randomized controlled trial
Source: Trials. 2019 May 29;20:304. doi: 10.1186/s13063-019-3363-y (PMC6542052; doi:10.1186/s13063-019-3363-y)
Supplement: Supplementary file 4 — Strategy for oxygen saturation (SpO2) decreasing. (DOC 28 kb) [file 13063_2019_3363_MOESM4_ESM.doc]

| Additional file 4. Strategy for SpO_2_ decreasing | | | | | | | | | |
| --- | --- | --- | --- | --- | --- | --- | --- | --- | --- |
| step | 1 | 2 | 3 | 4 | 5 | 6 | 7 | 8 | 9 |
| FiO_2_ | 0.5 | 0.6 | 0.6 | 0.7 | 0.7 | 0.8 | 0.8 | 1.0 | RM |
| standard PEEP group (cm H_2_O) | 5 | 5 | 4 | 4 | 3 | 3 | 2 | 2 | 2 |
| low PEEP ventilation group (cm H_2_O) | 3 | 3 | 4 | 4 | 5 | 5 | 6 | 6 | 6 |
| FiO_2_= Fraction of inspired oxygen; PEEP= Positive end-expiratory pressure; RM= Recruitment maneuver | | | | | | | | | |
