# Supplementary material for: Effects of intraoperative PEEP on postoperative pulmonary complications in patients undergoing robot-assisted laparoscopic radical resection for bladder cancer or prostate cancer: study protocol for a randomized controlled trial
Source: Trials. 2019 May 29;20:304. doi: 10.1186/s13063-019-3363-y (PMC6542052; doi:10.1186/s13063-019-3363-y)
Supplement: Supplementary file 5 — Definitions of study endpoints. (DOC 42 kb) [file 13063_2019_3363_MOESM5_ESM.doc]

| Additional file 5. Definitions of study endpoints. | | |
| --- | --- | --- |
| **Study endpoints** |  | Detailed description |
| **Pulmonary endpoints** |  |  |
| **Pneumonia** |  | Patients with altered or new pulmonary opacities on chest X-ray; patients should also meet at least two of the following criteria: (1) temperature ≥ 38.5℃ or < 36℃, (2) white blood cell (WBC) count > 12 x10^9^/L or < 4 x10^9^/L; (3) purulent sputum: new cough or difficulty breathing or previous coughing or difficulty breathing is further aggravated. |
| **Purulent sputum** |  | New cough or difficulty breathing or previous coughing or difficulty breathing is further aggravated. |
| **Hypoxemia** |  | Hypoxemia is defined as PaO_2_ < 60 mmHg or oxygen saturation (SpO_2_) < 90% on room air but responding to oxygen treatment (hypoventilation should be excluded). |
| **Severe hypoxemia** |  | Severe hypoxemia is recorded in cases when the patients require non–invasive or invasive mechanical ventilation. |
| **Suspected pulmonary infection** |  | The patient takes antibiotics and should meet at least one of the following criteria: (1) changed or new sputum, (2) changed or new pulmonary opacities on chest X–ray, (3) temperature greater than 38.3°C, and (4) WBC count > 12 x10^9^/L. |
| **Suspected pulmonary complications** |  | Patients display at least three of the following new findings: (1) cough, (2) increased secretions, (3) dyspnea, (4) chest pain, (5) temperature> 38°C, and (6) pulse rate> 100 beats per minute. |

| Continued Additional file 5. Definitions of study endpoints | | |
| --- | --- | --- |
| **Study endpoints** |  | Detailed description |
| **Extrapulmonary endpoints** |  |  |
| **SIRS** |  | SIRS are defined when meeting the following four criteria by the most deranged value recorded after surgery: (1) rectal or tympanic temperature > 38°C or <36°C (0.5°C will be added to the measured value when oral or other temperatures are used); (2) ventricular rate > 90 beats/min (excluding those who have a known medical condition or are receiving treatment that would prevent tachycardia); (3) respiratory rate> 20 breaths/min or a PaCO_2_< 32 mmHg or requiring mechanical ventilation; (4) WBC count >12 x 10^9^/L or < 4 x 10^9^/L. |
| **Sepsis** |  | Sepsis is recorded when meeting at least two SIRS criteria with a defined focus of infection. Defined infection is indicated in patients when they meet at least one of the following criteria: (1) an organism grown in blood or sterile site, (2) an abscess, (3) infected tissue (e.g., pneumonia, urinary tract, peritonitis, soft tissue, vascular infection, etc.). |
| **Severe sepsis** |  | Severe sepsis is recorded in a patient with sepsis who has at least one organ failure, hypotension or hypoperfusion. |
| **Septic shock** |  | Regardless of how adequate fluid resuscitation has been administered, the patient remains with sepsis-induced hypotension with the presence of perfusion abnormalities. |
| **SSI** |  | SSI defined as surgical site infection within 30 days after surgery; at least the incision has a purulent effluent; the incision drainage fluid or tissue culture results are positive, with pain or tenderness, local swelling, redness or fever. |
|  |  |  |

SIRS= Systemic inflammatory response syndrome; SSI= surgical site infection.
