# Supplementary figures and images for: Effects of intraoperative PEEP on postoperative pulmonary complications in patients undergoing robot-assisted laparoscopic radical resection for bladder cancer or prostate cancer: study protocol for a randomized controlled trial
Source: Trials. 2019 May 29;20:304. doi: 10.1186/s13063-019-3363-y (PMC6542052; doi:10.1186/s13063-019-3363-y)

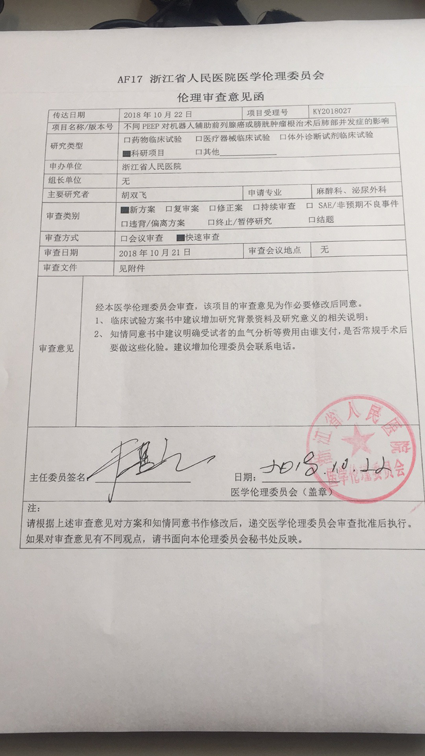

Supplement: Supplementary file 6 — Appendix Figure study institutional review board (IRB) in original language. (TIF 390 kb) [file 13063_2019_3363_MOESM6_ESM.tif]

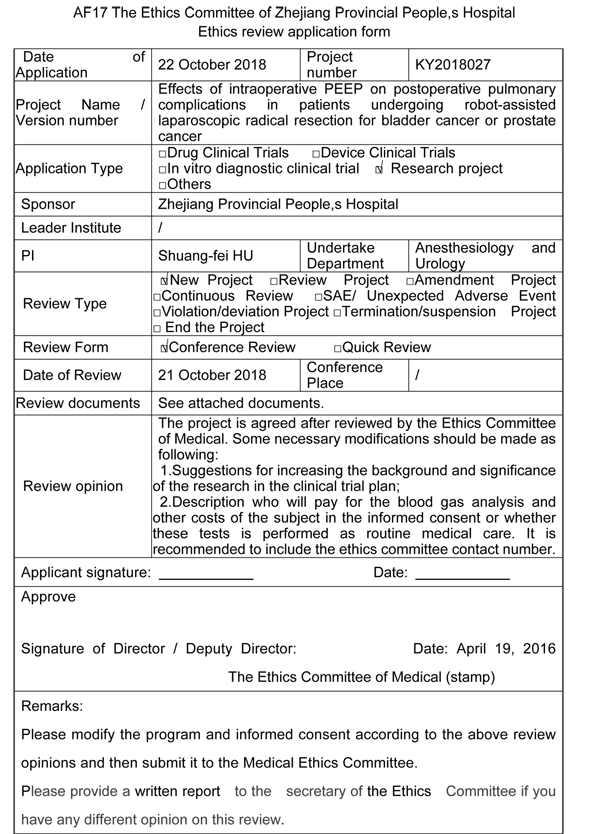

Supplement: Supplementary file 7 — Appendix Figure study institutional review board (IRB) in English. (TIF 401 kb) [file 13063_2019_3363_MOESM7_ESM.tif]
